# Development of a decision model for the selection of exoskeletons for application in automotive production plants

**DOI:** 10.1371/journal.pone.0333420

**Published:** 2025-10-14

**Authors:** Woun Yoong Gan, Raja Ariffin Raja Ghazilla, Hwa Jen Yap, Suman Selvarajoo, Zhang Jieshu

**Affiliations:** Department of Mechanical Engineering, Faculty of Engineering, Centre for Sustainable and Smart Manufacturing (CSSM), University of Malaya, Kuala Lumpur, Malaysia; King Fahd University of Petroleum & Minerals, SAUDI ARABIA

## Abstract

This paper presents the development of a decision model for the selection of exoskeletons for application in automotive production plants. The decision model consisted of three stages: (1) Human Factor-Failure Mode and Effect Analysis (HF-FMEA), (2) augmentation analysis, and (3) Preference Ranking Organization Method for Enrichment Evaluation (PROMETHEE). This decision model is called the Decision Model for Exoskeleton Selection in the Automotive Industry (DMESAI). An industrial case study was conducted with 13 experts from the automotive industry to test and verify the usability of the DMESAI. The findings suggest that the DMESAI is feasible to determine the need for exoskeletons in automotive production processes, narrowing the suitable types of exoskeletons for specific tasks, and addressing user’s preferences.

## Introduction

Exoskeletons are wearable devices that assist humans to perform various routine and nonroutine tasks. Exoskeletons consist of mechanical frames and joints that protect and improve human motion. Exoskeletons can be classified as mechanical and electrical, as well as active and passive. The application of exoskeletons varies depending on the needs of the user such as rehabilitation, assisting the user with disability, performing heavy and repetitive tasks, and even rescue missions. However, exoskeletons face ergonomic issues due to their weight and discomfort, as well as restrictions in motions. To date, there are limited studies on the development of a decision model for the selection of exoskeletons for specific tasks. Therefore, in this study, a decision model was developed for the selection of suitable exoskeletons for use in automotive production plants based on ergonomic risks and task requirements. Several methods were studied and integrated as a decision flow model to identify the appropriate exoskeleton based on the needs of the user. Human Factor- Failure Mode and Effect Analysis (HF-FMEA) and augmentation analysis were carried out before implementing the Preference Ranking Organization Method for Enrichment Evaluation (PROMETHEE).

## Literature review

### Exoskeletons

Currently, exoskeletons are mainly used in medical and industrial applications. Exoskeletons are used in the medical industry to assist people with disabilities and impairments with daily living and rehabilitation, whereas exoskeletons are used in industries to assist workers in performing daily tasks. There are two types of exoskeletons: active and passive. Active exoskeletons consist of one or more actuators that increase human strength and assist in actuating the joints. These actuators can be pneumatic muscles, electric motors, hydraulic actuators, or other types [1]. A strictly passive system uses materials, springs, or dampers that can store energy captured by human motion and use it as needed to support a posture or motion, without implementing actuators. Exoskeletons can also be identified by the bodily parts they support: full-body exoskeletons support both the upper and lower extremities, upper-body exoskeletons support the upper extremities, and lower-body exoskeletons support the lower limbs. Some single-joint exoskeletons have also been designed and developed by researchers. Lower-body exoskeletons are commonly used as rehabilitation aids and improve the quality of life for people with disabilities. These exoskeletons are also the most successful products of recent times [2]. Recently, lower-body exoskeletons have been primarily used for rehabilitation and mobility enhancement of patients with conditions such as cerebral palsy or spinal cord injuries [3].

### Ergonomics

The working environment of workers influences their performance, productivity, and health. Therefore, it is essential to provide a suitable workstation and the necessary support equipment for workers to attain the desired output while ensuring minimal impact to their physical and mental health [4]. A good workstation should be ergonomically designed to minimize work-related hazards [5]. This can be achieved by performing macro ergonomic analysis of the manufacturing system by experts on various aspects such as medicine, sociology, psychology, technical sciences, and business to attain an optimum workstation design. Workers often experience health issues due to poor ergonomics, which often leads to work-related musculoskeletal disorders (WMSDs) [6]. They tend to experience issues with their joints, shoulders, back, neck, hip, elbows, knees, and multiple areas due to prolonged exposure to poor ergonomic conditions. One of the activities that results in injuries is the welding process, which demands continuous physical effort from the operators [7]. Therefore, it is necessary for industries to assess workplace design and practices to minimize ergonomic risks in the workplace while making necessary improvements from time to time [8].

### Automotive production process

Manufacturing operations in the automotive sector begin with the selection of various materials for the automotive specifications and this requires engineers to conduct extensive research on the design and range of components [9,10]. In most cases, the automotive industry outsources parts to suppliers and focuses on the final assembly, though some processes such as stamping may be performed in-house to ensure quality and precise geometry [11,12]. The major process is basically the same for passenger and commercial vehicle assembly, and only certain processes vary depending on the product requirements. The process generally involves a few key departments such as press shop, body shop, power train shop, painting shop and assembly shop, where each shop plays a distinct role [13,14]. In addition, there are many subassembly stations involved to assemble the various components of a vehicle. Besides the processing department, the automotive production process also involves the logistics department, quality control department, repair department, and in some cases, machining department.

### Current status of exoskeletons in Malaysia

In Malaysia, the development of most exoskeletons is still in the research and prototype stages, led by universities with limited commercial application. For example, [15] developed a soft hand exoskeleton using a rubber actuator for stroke patients [16] proposed a brain-controlled full-body exoskeleton to address the issues of weight and flexibility. [17] conducted a mechanical study of lower limb exoskeleton design based on typical Malaysian heights for leg rehabilitation These studies are mainly focused on rehabilitation. As a developing nation, Malaysia still faces challenges to provide optimal rehabilitation for individuals with physical disabilities, and exoskeletons offer potential to improve unsupervised therapy and reduce rehabilitation costs [18]. Currently, 24 hybrid assistive limb units are used by the Social Security Organization at the Cybernics Center in the Tun Abdul Razak Rehabilitation Centre (TRRC) in Ayer Keroh, and Weston Robot from Singapore has conducted trials on exoskeletons in Vesuvius Malaysia for loading and packing tasks. Meanwhile, the industrial application of exoskeletons remains limited, and only a few studies have been carried out such as [19] passive sit–stand exoskeleton for workers in electronics and semiconductor sectors, which received positive user feedback, and Tahmida’s hybrid exoskeleton, which reduced muscle strain during oil palm harvesting by up to 23% [20]. Several studies have also addressed the control systems, which is a major challenge in exoskeleton development in order to enhance the performance of exoskeletons [21–23].

### Decision model

The process of selecting an appropriate exoskeleton for a given application is challenging, and users have little assistance to make decisions. [24] proposed a framework for selecting an occupational exoskeleton that focused on categorizing various criteria on tasks, workplace, user and human–machine interface. [25] introduced ExoMatch, which matched key traits of exoskeletons with workplace requirements by analyzing data from both the technology and production environment to define the accurate selection criteria. Ralfs, Hoffmann [26] proposed a seven-phase model for evaluating exoskeletons: characterization, preparation, predevaluation, core evaluation, post evaluation, analysis, and reflection. The model views exoskeleton uses as an interaction between the user, device, and work environment. The model begins with assessing the workplace scenario and setting up the evaluation environment. Golabchi, Riahi [27] proposed a framework that enabled the workplace to systematically assess and implement industrial exoskeletons, where the framework consisted of six stages: feasibility evaluation, task selection, exoskeleton selection, implementation logistics, trial phase, and long-term adoption. Based on the literature review, there are some useful methods for exoskeleton selection such as structured database filtering, ergonomic assessment tools, and early-stage evaluation frameworks. Each of these methods offers strengths, including clear attribute classification, preselection algorithms, and user-centered evaluation processes. Based on these advantages, a few of these methods were integrated and refined in this study to develop the DMESAI.

### Selection of tools for the DMESAI

The DMESAI is a model developed to fulfill the following objectives: (1) to identify the risks that occur among workers in the industry, (2) to match the selected high-risk tasks with the exoskeleton characteristics, (3) to select the body part that is most affected by the task, and (4) to select the suitable exoskeleton for a particular task. For the task assessment tool, HF-FMEA was chosen over other ergonomic tools such as Rapid Upper Limb Assessment (RULA) and Rapid Entire Body Assessment (REBA) [28] as it can identify high-risk postures and quantify the severity, occurrence, and detectability of ergonomic failures in different production tasks in the production line, enabling a prioritization of tasks for exoskeleton implementation. To match the exoskeletons with the task requirements, augmentation analysis was introduced, built on the fundamental characteristics of exoskeletons and a rating system linked to the workplace conditions. This approach ensures that tasks with high ergonomic risks are appropriately matched to the exoskeletons. For the exoskeleton selection method, PROMETHEE was selected over other multicriteria decision model (MCDM) methods such as Analytic Hierarchy Process (AHP) and Technique for Order of Preference by Similarity to Ideal Solution (TOPSIS) due to its ability to handle both qualitative and quantitative criteria with clear visual outputs, making it more intuitive for industry practitioners [29].

## Development of the DMESAI

The DMESAI was developed to facilitate industry practitioners to select a suitable exoskeleton for a particular task. The DMESAI involves several processes that allow users to identify human-related risks in the production environment and select a suitable exoskeleton based on the user’s preferences. The first step of the DMESAI involved performing the HF-FMEA, which was used to assess the ergonomic risks. Next, augmentation analysis was carried out to evaluate the task requirements, and finally, PROMETHEE was used to identify body parts prone to fatigue as a result of performing tasks and to enable users to select the most suitable exoskeleton.

### HF-FMEA

The first stage of the DMESAI involved conducting the HF-FMEA, where the users identified the current or potential ergonomic risks within different production stations. The HF-FMEA involved examining the automotive production flow and identifying the three factors used to assess the risks, namely, severity (SEV), occurrence (OCC), and detection (DET). The description of the ratings used in HF-FMEA is shown in Table 1. These factors were then used to determine the risk priority number (RPN) for different stations.

**Table 1:** Description of the ratings used in the HF-FMEA.

	1	2	3	4
Severity (SEV)	Minor	Moderate	Major	Catastrophic
	Minimal discomfort, unlikely to cause long-term health effects	Possible short-term discomfort, but unlikely to cause serious health issues	Can lead to WMSDs over time	Task causes severe pain, potential injuries, and immediate risk of musculoskeletal injuries
Occurrence (OCC)	Rare	Uncommon	Occasional	Frequent
	Occurs infrequently, only a few times a week or less	Occurs periodically, a few times a day or several times a week	Occurs frequently throughout the day, possibly multiple times in an hour	Occurs continuously, with very little to no breaks in between
Detection (DET)	High detection (easy to identify)	Medium detection	Low detection	Rare detection (almost not detectable)
	Ergonomic issues are promptly identified and addressed through regular inspections and feedback systems	Ergonomic issues are noticed and addressed during assessments or when individuals report discomfort	Ergonomic issues are often only addressed after individuals experience discomfort or when they report problems	Ergonomic issues are rarely detected, and individuals continue to experience discomfort

HF-FMEA was carried out to evaluate the human factor risks in automotive production tasks. First, human factors such as ergonomic strains and human errors and the potential failure modes of a production task were identified. Next, each risk was assessed using SEV, OCC, and DET. Direct observations of few production stations were performed to gather data, with emphasis on operations involving significant manual labor. This makes it possible to prioritize ergonomic solutions using the determined RPN and conduct a methodical study of human factor risks.

### Augmentation analysis

Augmentation analysis was performed to identify the suitability of exoskeletons for the high-risk tasks determined from the HF-FMEA. The augmentation analysis included studying the exoskeleton characteristics and production process. The augmentation analysis bridged the gap between the task requirements and exoskeleton capabilities. The augmentation analysis involved evaluating the production tasks and matching the tasks with the exoskeleton characteristics, and consisted of five sections, namely, task analysis, working environment, current equipment used to perform the tasks, current risks, and the level of acceptance of exoskeleton technology among the workers. The augmentation analysis matched the tasks with the possibility of implementing exoskeletons in the existing production environment. Each question addressed the key criteria and the responses were binary, with ‘Yes’ indicating the presence of the attribute and ‘No’ indicating its absence. Table 2 shows the description of each question in relation to the suitability of exoskeletons. The questionnaire consisted of 17 questions, where each was assigned a score of 1. Based on the total score (ranging from 1 to 17), different suggestions were made for exoskeleton suitability. However, these were merely suggestions, and the user can make their own decision based on their preferences.

**Table 2:** Questionnaire used for the augmentation analysis.

No.	Group	Question	Rating
Q1	Task analysis	Does the task require frequent movements within the station and in between stations? (i.e., walking)	Yes: 0 No: 1
Q2		Does the task require repetitive motions?	Yes: 0 No: 1
Q3		Does the task involve lifting heavy objects or equipment?	Yes: 0 No: 1
Q4		Does the task require awkward postures (e.g., bending, twisting, squatting, laying down, working above shoulder height, etc.)?	Yes: 0 No: 1
Q5		Does the task demand high precision or complex movements and skills?	Yes: 0 No: 1
Q6		Does the task require dynamic frequent changes in direction or adjustments in posture?	Yes: 1 No: 0
Q7		Does the task require holding on a position/ posture for extended periods? (e.g., standing, static posture, etc.)	Yes: 1 No: 0
Q8	Working environment	Is the workstation sufficient to minimize WMSDs among workers?	Yes: 0 No: 1
Q9		Is the workspace sufficient for movement with exoskeletons?	Yes: 1 No: 0
Q10		Is the task performed in a normal working environment (i.e., room temperature, neither extremely hot nor extremely cold)	Yes: 1 No: 0
Q11		Is the task free from specific safety hazards (e.g., chemical exposure, risk of falling objects, high voltage, etc.)?	Yes: 1 No: 0
Q12	Current equipment	Does the task require contact with machinery or robots?	Yes: 0 No: 1
Q13		Does the task involve the use of tools, complex equipment, or personal protective equipment that may affect the use of exoskeletons?	Yes: 0 No: 1
Q14		Does the task involve the use of vibration equipment, impact tools, etc. that may lead to fatigue and WMSDs?	Yes: 1 No: 0
Q15	Risks	Does the task lead to physical exhaustion and pose a high risk for WMSDs?	Yes: 1 No: 0
Q16		Do the workers remain performing the same tasks even if they pose a high risk for WMSDs?	Yes: 1 No: 0
Q17	Acceptance	Do the workers accept wearable devices for working?	Yes: 1 No: 0

### PROMETHEE for body part selection

PROMETHEE, as part of MCDM, was then developed using Visual PROMETHEE software. The outcomes generated from the Visual PROMETHEE software were the PROMETHEE rankings. Two PROMETHEE rankings were computed, namely, PROMETHEE I and PROMETHEE II. PROMETHEE I Partial Ranking is based on the computation of two preference flows (Phi+ and Phi-). It allows the incomparability between actions when both Phi+ and Phi- preference flows give conflicting rankings. In contrast, PROMETHEE II Complete Ranking is based on the net preference flow (Phi). PROMETHEE II is more suitable for this study as the preferences are well-quantified, allowing straightforward aggregation into a net preference flow [30].

The first stage of PROMETHEE involved identifying the criteria for the risk of body fatigue when performing the production tasks selected from previous tasks. The body parts and criteria were identified to analyze the risks. In this study, only five body parts were covered, which were the arms, back, shoulders, knees, and waist due to the availability of the exoskeleton models in the market. The user was required to rate the level of fatigue experienced in different body parts while performing the tasks selected from previous tools. The purpose of this step was to identify the body part that may benefit from an exoskeleton tailored for the particular task. The criteria for the body part selection are shown in Table 3.

**Table 3:** Criteria for body part selection.

	Criteria	Description	Rating by users
1	Awkward posture	This criterion identifies the awkward postures associated with five different body parts while performing a task.	Very low to very high
2	High force and load	This criterion evaluates tasks that involve high force and load requirements, such as lifting, pushing, pulling, or carrying heavy objects.	Very low to very high
3	Repetitive and frequent motions	This criterion examines the tasks that require repetitive or frequent motions, such as repeated lifting, assembly, or tool operations.	Very low to very high
4	Long-operation posture and duration	This criterion focuses on the tasks that require maintaining a specific posture or position for extended periods, such as prolonged standing, sitting, or holding a static posture.	Very low to very high
5	Vibrations	This criterion evaluates the tasks involving exposure to vibrations, such as using power tools or operating machinery.	Very low to very high
6	Fatigue and injury	This criterion identifies whether the selected body part experiences fatigue or injury while performing the task.	Yes or no

### PROMETHEE for exoskeleton selection

The second stage of PROMETHEE, which was also the last stage of the DMESAI, involved the selection of a suitable exoskeleton using Visual PROMETHEE software. The 12 criteria for exoskeleton selection were identified through the literature review and were filtered to ensure that each criterion was independent of others [31–52], as shown in Table 4. In addition, in the authors’ previous study [53], all of the selected criteria were reviewed by 10 experts from the automotive industry to rate their weights using AHP. The weight of each criterion is also presented in Table 4. The criteria were rated by the experts based on their experience in implementing a new technology in their workplace. The preference function was set based on the characteristics of the data and decision context. However, the weight and preference function of each criterion served as a general reference and can be adjusted by the user in different cases based on their perspective. For certain criteria, such as the cost of purchasing an exoskeleton, parameters such as indifference threshold (q) and preference threshold (p) were defined by the users based on their preferences.

**Table 4:** Criteria for exoskeleton selection.

	Criteria	Description	Rating by users	Weight (%)	Preference function
1	Purchasing costs of exoskeletons	This criterion allows users to specify their preference for purchasing costs when comparing exoskeleton models.	Amount	5.29	V-shape
2	Increase user’s strength, endurance, and work quality	This criterion enables users to evaluate exoskeleton models based on their ability to enhance the user’s strength, endurance, and work quality.	Very low to very high	6.26	Usual
3	Reduction of WMSDs and fatigue	This criterion evaluates the exoskeleton models based on their effectiveness in reducing WMSDs and fatigue.	Percentage of fatigue reduction	10.52	Level
4	User-friendliness, comfort, and safety of exoskeletons	This criterion assesses the exoskeleton models based on their user-friendliness, comfort, and safety.	Very low to very high	8.90	Usual
5	Durability and reliability of exoskeletons	This criterion evaluates the durability and reliability of exoskeleton models.	Very low to very high	8.09	Usual
6	Degree of freedom of exoskeletons (user flexibility)	This criterion assesses the exoskeleton models based on their degree of freedom, which reflects the level of flexibility and range of motions they allow for the user.	Very low to very high	5.20	Usual
7	Suitability of exoskeletons for production requirements	This criterion evaluates the exoskeleton models based on their suitability to fulfil specific production requirements.	Very low to very high	5.98	Usual
8	Weight and size of exoskeletons	This criterion assesses the exoskeleton models based on their weight and size.	Weight (kg)	4.97	V-shape
9	Power source and usage interval (battery life)	This criterion evaluates the exoskeleton models based on their power source type and battery life.	Very low to very high	5.19	Usual
10	Maintenance and operation costs of exoskeletons	This criterion evaluates the exoskeleton models based on their maintenance and operation costs.	Very low to very high	14.69	Usual
11	Ease of maintenance and after-sales support	This criterion assesses the exoskeleton models based on their ease of maintenance and the quality of after-sales support.	Very low to very high	18.91	Usual
12	Training and competency requirements	This criterion evaluates the exoskeleton models based on their training and competency requirements.	Very low to very high	6.00	Usual

### Selection of exoskeleton model

The final stage of the DMESAI involved selecting a suitable exoskeleton model available in the market for a particular automotive production task. In this study, the exoskeleton models for industry application available in the market were selected to use as selection options based on the user’s preference criteria. The final exoskeletons covered in this study were those that matched most of the selection criteria, which were arm-and-lumbar exoskeletons, back exoskeletons, lower-body exoskeletons, shoulder exoskeletons, and shoulder-and-elbow exoskeletons, as shown in Table 5. However, due to the limited availability of data provided by the exoskeleton manufacturers, some of the data were not provided in this study. In these cases, in order to maintain the objectivity of the PROMETHEE ranking process, the missing values were left blank and excluded from the calculation of the preference flows for that specific alternative, ensuring that only verified and reliable data contributed to the decision-making process. The framework enabled missing data to be added in later once available, and the ranking results can be recalculated based on the updated dataset.

**Table 5:** Exoskeleton models.

Type of exoskeleton	Model name
**Arm, Back**	ExoARMS
	Muscle upper
	Hyetone STRONG HANDS
**Back**	Laevo V2.6
	Exyone Back Lite
	Muscle suit EXO Power
	ExoBack (RB3D)
	Seismic Suit
	HAPO SD
**Shoulder + Elbow**	HAPO front
	HAPO UP
**Chairless Chair**	Noonee Chairless Chair 2.0
	Archelis for Industrial Use
	Sit Anytime PHEL1-Squat Assisted Exoskeleton
**Shoulder**	Exyone Shoulder
	Ekso EVO
	DeltaSuit (Auxivo)
	IX SHOULDER AIR
	PLUM

## Application of the DMESAI

**Fig 1:**
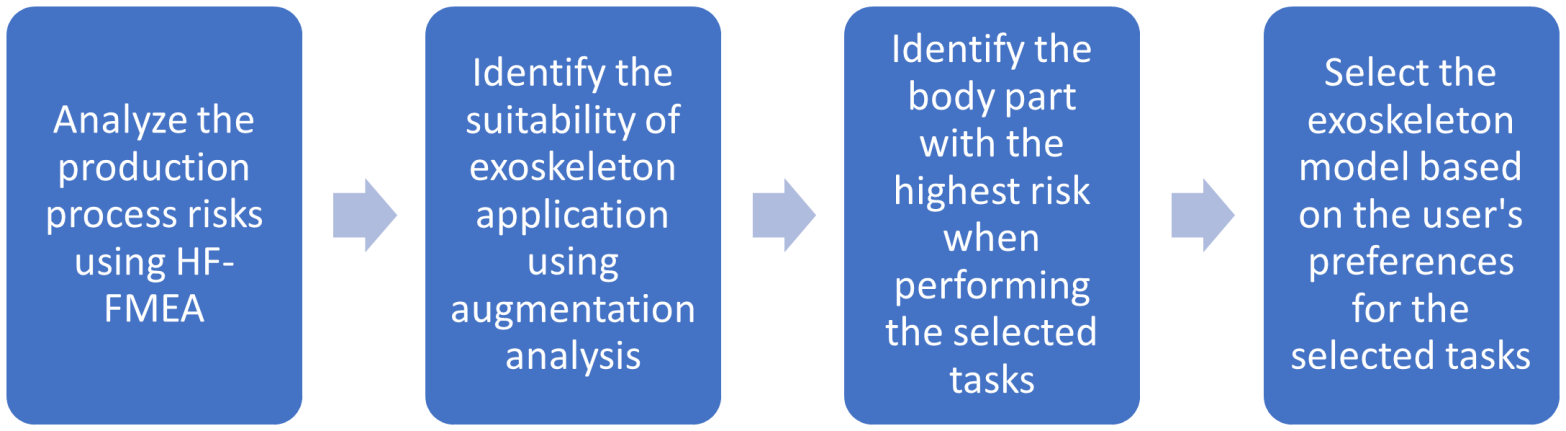
Flow chart of DMESAI.

This section covers the trial operation of the DMESAI based on the tasks of the authors’ previous study [54]. The tasks involved welding the side structure frame for bus assembly and seat installation for passenger car assembly to analyze the risk and selection of the exoskeleton. The flow chart of the DMESAI is shown in Fig 1.

### HF-FMEA

The tasks of welding the side structure involved welding the side structure frame, grinding, and finishing the welding surface. While performing the welding tasks, the workers were required to bend, kneel, and work above shoulder height, whereas for grinding, the workers were required to bend the body to grind. Both of these tasks were performed by the workers manually. The RPNs for the tasks were both rated 32. For the seat installation tasks, the workers were required to carry the seat into the car body and conduct the assembly. The workers were required to bend their body to carry the seat into the car, and assemble the part in a bending and kneeling posture. The RPNs for the car seat assembly were 36 and 24. Table 6 provides a detailed breakdown of the HF-FMEA based on the tasks (welding of the side structure frame for bus assembly and seat installation for passenger car assembly), including the risk categories and their associated RPNs.

**Table 6:** HF-FMEA for welding of the side structure frame for bus assembly and seat installation for passenger car assembly.

Main Tasks (station)	Sub-task	Ergonomic posture (working posture)	Automated/ manual	Potential Ergonomics Risks	SEV	Sources of risk	OCC	Risk Control Measures	DET	RPN	Recommended controls
Welding (bus) – side structure frame	Welding the side structure frame of the bus assembly	Welding while bending, kneeling, and working above shoulder height	Manual	Awkward postures	4	Welding postures	4	Alternative tasks	2	32	Automation/ exoskeletons
	Grinding and finishing	Bend to grind the surface of the welded parts	Manual	Awkward postures Repetitive motions Exposure to vibrations Heavy lifting and handling Dust and fumes	4	Long hours of operation to grind the structure, involving different postures	4	Alternative tasks	2	32	Automation/ exoskeletons
Assembly – seat installation	Carry car seat into the car body	1. Bend body to carry car seat into the car body	Manual	Heavy lifting and handling Awkward postures Confined spaces	3	Pack area in the car body and size of the seats	4	Audit every 3 months	3	36	Exoskeletons/ ergonomic devices
	Car seat assembly	Bending and kneeling to fasten the car seat in place	Manual	Heavy lifting and handling Awkward postures Exposure to vibrations Confined spaces	3	Pack area in the car body and size of the seats	4	Audit every 3 months	2	24	Exoskeletons/ ergonomic devices

### Augmentation analysis

Among the tasks identified from the HF-FMEA, the main tasks with the highest RPNs were selected for augmentation analysis, where T1 denotes welding the side structure frame for bus assembly, T2 denotes grinding and finishing, T3 denotes carrying the car seat into the car body, and T4 denotes car seat assembly. The results for T1, T2, T3, and T4 were 12, 15, 12, and 13, respectively. The augmentation analysis results are presented in Table 7. The results indicated that all of the tasks were highly suitable for the application of exoskeletons.

**Table 7:** Augmentation analysis.

Main Tasks	Task	Q1		Q2		Q3		Q4		Q5		Q6		Q7		Q8		Q9		Q10		Q11		Q12		Q13		Q14		Q15		Q16		Q17		marks
		Yes – 0	No – 1	Yes – 1	No – 0	Yes – 1	No – 0	Yes – 1	No – 0	Yes – 0	No – 1	Yes – 1	No – 0	Yes – 1	No – 0	Yes – 0	No – 1	Yes – 1	No – 0	Yes – 1	No – 0	Yes – 1	No – 0	Yes – 0	No – 1	Yes – 0	No – 1	Yes – 1	No – 0	Yes – 1	No – 0	Yes – 1	No – 0	Yes – 1	No – 0	
Welding (bus) – side structure frame	T1	✓		✓		✓		✓		✓		✓		✓			✓	✓		✓		✓		✓		✓		✓		✓		✓			✓	12
	T2	✓		✓			✓	✓			✓	✓		✓			✓	✓		✓		✓			✓		✓	✓		✓		✓		✓		15
Assembly – car installation	T3	✓		✓		✓		✓		✓		✓			✓		✓		✓	✓		✓			✓		✓	✓		✓		✓			✓	12
	T4		✓	✓			✓	✓		✓		✓		✓			✓		✓	✓		✓			✓		✓	✓		✓		✓			✓	13

### Body part selection

**Fig 2:**
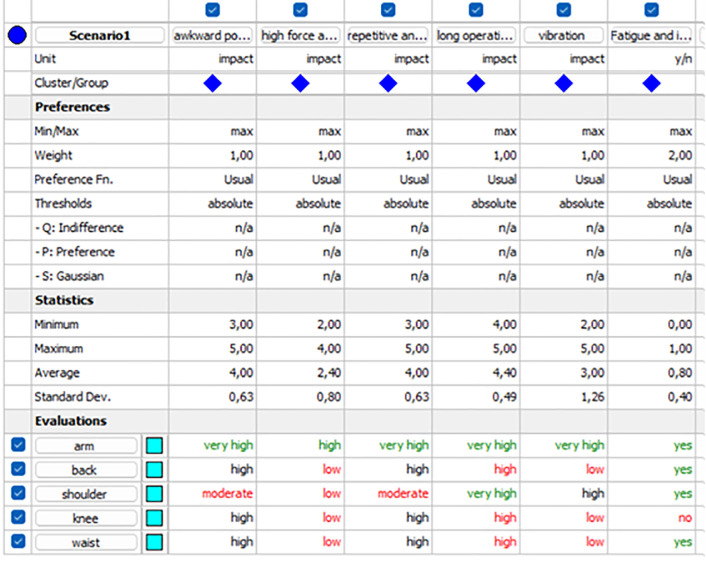
Evaluation of selected body parts.

**Fig 3:**
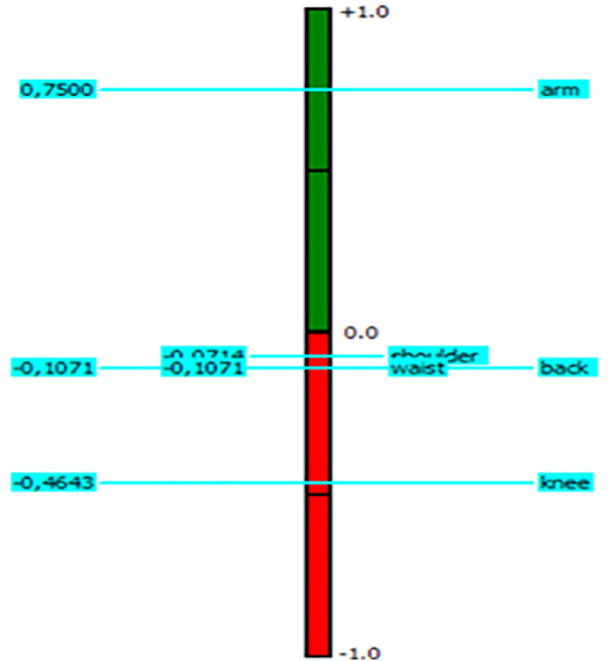
Complete ranking of the selected body parts.

Among all of the selected tasks, T2 (grinding and finishing) was rated as the most suitable task for exoskeleton application. Hence, this study is focused on analyzing the selection of exoskeletons for T2. Visual PROMETHEE software was used to select the body parts and suitable exoskeletons. The selected body part for T2 was the arm, with a phi value of 0.75. Fig 2 shows the evaluation of the selected body parts according to the selection criteria for T2, while Fig 3 shows the complete ranking of the selected body parts for T2.

### Exoskeleton selection

**Fig 4:**
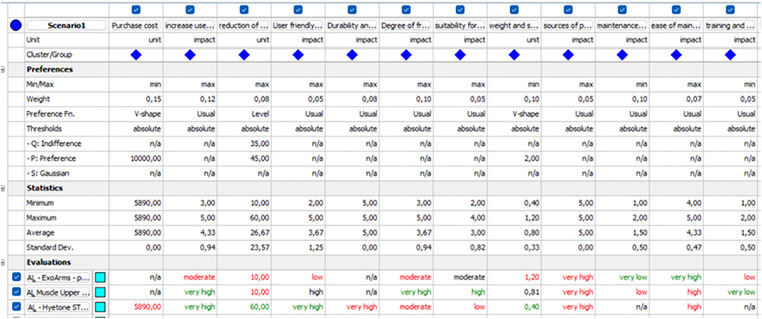
Exoskeleton selection criteria.

**Fig 5:**
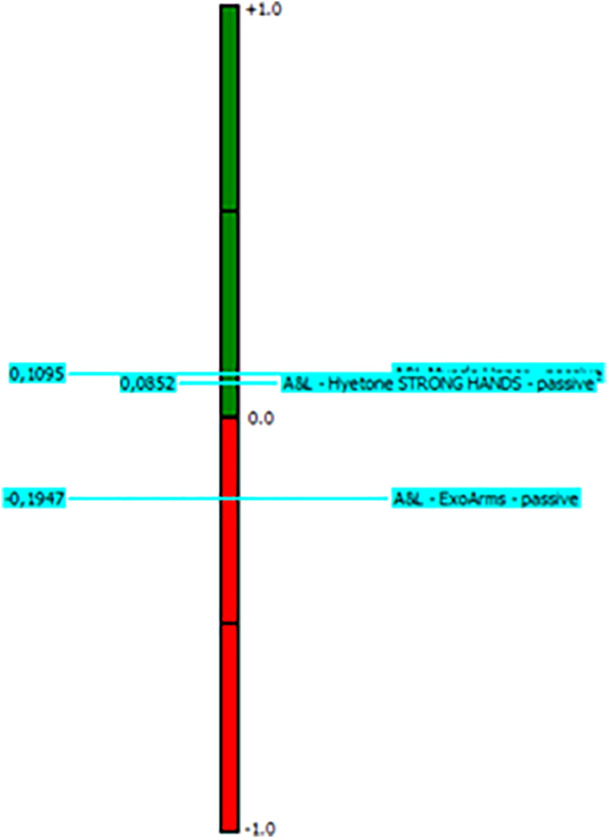
Result of the selection.

The exoskeletons were selected based on the body parts selected using Visual PROMETHEE software, where arm exoskeletons were selected for T2. The settings of the exoskeleton selection criteria are shown in Fig 4 and the results of the selection are shown in Fig 5. In this study, the selected exoskeleton for the grinding task was Muscle Upper, with a phi value 0.1095.

### Industrial case study

The DMESAI was tested by 13 experts from various automotive assembly industries in Malaysia to determine the usefulness of the DMESAI and obtain feedback from them. The DMESAI was also tested to assess the impact of the decision model to the industries and determine whether the model was successful. The experts recruited for the industrial case study were from the passenger car assembly industry, bus assembly industry, and truck assembly industry. Before each interview session began, participants were informed that the session would be recorded and were provided with a clear explanation of the study’s purpose, procedures, and their rights, including confidentiality, and the right to withdraw at any time. Verbal consent was then explicitly obtained and documented by recording the participant’s spoken agreement at the beginning of each session. A trained research assistant, and in some cases management representative from the participant’s organization was present as a witness during the consent process to ensure transparency and compliance. The research ethics, including consent form, method of research process, was reviewed and approved by the Universiti Malaya Research Ethics Committee, under approval reference number UM.TNC2/UMREC_4200. Each expert was asked to propose 3–4 tasks with risks in their production line. Among the proposed tasks, only the tasks with the highest RPNs obtained from HF-FMEA were considered as the most critical tasks and the exoskeleton selection was proceeded for each case. Following the sequence of the DMESAI described in the previous section, the results of the evaluation conducted with 13 experts are summarized in Table 8.

**Table 8:** Outcomes of the industrial case study.

Expert	Industry	Task	Type of exoskeleton	Exoskeleton model	Phi value
1	Passenger car assembly	Applying adhesive to the car body	Arm	ExoARMS	0.0381
		Applying door sealant	Arm	ExoARMS	0.0381
2	Passenger car assembly	Mounting of instrument panel	Back	ExyONE Back Lite exoskeleton	0.2144
		Wiring connection of instrument panel	Arm	Muscle Upper exoskeleton	0.1679
3	Bus assembly	Spraying side panel	Arm and shoulder	Hyetone STRONG HANDS exoskeleton	0.2387
4	Bus assembly	Carrying seat to the car body	Back	RB3D ExoBack exoskeleton	0.1340
5	Bus assembly	Welding of the hollow bars	Arm and shoulder	Hyetone STRONG HANDS exoskeleton	0.3341
		Grinding the surface	Arm and shoulder	Hyetone STRONG HANDS exoskeleton	0.3326
6	Bus assembly	Carrying windshield and installation	Shoulder	ExyOne shoulder exoskeleton	0.2129
7	Passenger car assembly	Spot welding underbody subframe assembly	Back	ExyONE Back Lite exoskeleton	0.0721
		Spot welding for roof assembly	Back	ExyONE Back Lite exoskeleton	0.0721
8	Truck assembly	Carrying and lifting battery	Back	ExyONE Back Lite exoskeleton	0.1249
9	Bus assembly	Installing propeller shaft	Back	ExyONE Back Lite exoskeleton	0.0883
10	Passenger car assembly	Door subassembly	Arm	Muscle Upper exoskeleton	0.1149
11	Truck assembly	Carrying dashboard to the body and tightening	Back	ExyOne Back Lite exoskeleton	0.0930
12	Passenger car assembly	Mounting axle	Back	RB3D ExoBack exoskeleton	0.0630
13	Passenger car assembly	Installing the windshield	Arm and shoulder	Hyetone STRONG HANDS exoskeleton	0.2528

### Feedback from the industrial experts

After the data were collected from the 13 experts, the experts commented on the DMESAI and their feedback was recorded in the feedback forms. All of the experts reported significant improvements in their ability to select a suitable exoskeleton for a specific task using the DMESAI. The DMESAI provided a systematic and comprehensive framework, enabling better alignment of the task requirements with the exoskeleton features. This structured approach enhanced decision-making efficiency and understanding of exoskeleton applicability, particularly in mitigating risks such as fatigue and injuries. The experts highlighted that the evaluation process offered clear insights on matching exoskeletons with tasks based on their risks and production needs, allowing for more accurate and practical selections. The model also facilitated adjustments to meet specific task requirements, affirming organizational confidence in selecting the right product, and underscored the importance of customization for diverse applications. Overall, the evaluation increased their interest in exoskeleton technology and its potential benefits for production environments.

## Discussion

In this study, the DMESAI was developed to provide a structured approach to select a suitable exoskeleton for different tasks in the automotive production environment based on the task requirements and risks. The DMESAI involved HF-FMEA, augmentation analysis, and selection of body parts and exoskeletons using PROMETHEE, which provided a systematic model to evaluate the risks faced in different production processes and to identify a suitable exoskeleton that matched the user’s preferences. The findings showed that the DMESAI was capable of selecting a suitable exoskeleton model based on the user’s preferences. Compared with the single MCDM method, the DMESAI offered a sequence of evaluations to analyze the risks faced by workers in performing various tasks in different production stations. This provides insights into which tasks require exoskeletons and the actions that can be taken to reduce the risks. By inputting the user’s preferences on different criteria, the exoskeleton model available in the market can be selected with ease. The DMESAI was tested based on the tasks recommended by industry experts from the authors’ previous study. The DMESAI was also tested by performing a case study with 13 industry practitioners. The results showed that the DMESAI was able to identify the risks of the production tasks and select a suitable exoskeleton based on the user’s preferences. However, in this study, there were limited data available for some exoskeleton models in the market. The models with limited data affected the selection process. The main challenge identified through this study was the complexity of setting the preferences and identifying the risks in different production tasks, as it required the users to have some level of exposure to adjust the selection parameters effectively. Table 9 compares the DMESAI framework with other decision models proposed in previous studies, along with the advantages of other methods. The DMESAI provides a sequential structure that combines risk analysis and decision-making into a continuous decision model framework. Through an industrial case study, the DMESAI showed that it can be scaled to different factory layouts and company sizes, as its framework relies on user-defined inputs regarding production processes and ergonomic risk factors, rather than being limited to a single industrial configuration. The DMESAI can be integrated into digital platforms such as apps, which is a direction for future research. Even though the current DMESAI focuses on identifying risks from general production processes, integrating physiological or biomechanical data (e.g., electromyography, motion capture) into the HF-FMEA can also be carried out to improve the decision model in future work. Once digitized, the exoskeleton database of the DMESAI can be continuously updated as new products enter the market, and with slight adaptations to account for industry-specific characteristics, the DMESAI can also be applied to other fields such as construction, logistics, and healthcare.

**Table 9:** Comparison of the DMESAI with other decision models.

Reference	Selection process	Methods used	Type of framework
**Drees et al. (2023)**	1. Identified 99 selection criteria from the literature.	Literature synthesis, checklist filtering	Criteria-based selection framework
	2. Grouped criteria based on human and task-centered dimensions (Task & Workplace, User, Human–Machine Interface) based on the Political, Economic, Socio-Cultural, Technological (PEST) framework.		
	3. Filtered exoskeletons using categorized attributes (e.g., task compatibility, comfort, cost).		
	4. Ranked or prioritized exoskeletons based on weighted criteria (future integration with multicriteria decision analysis was suggested).		
**Dahmen and Constantinescu (2020)**	Developed database Database containing 140 exoskeletons with detailed attributes	Holistic methodology, workplace observations, iterative matching	Planning and integration framework
	Attribute classification General, technical features, compatibility, legal, pros and cons, costs Use case data attributes: project state, workplace, ergonomics, and environment		
	ExoScore: A scoring system was developed to preliminarily rate each exoskeleton. Attributes (e.g., maturity, cost) were assigned weighted factors depending on goals		
	ExoMatch: Matching rules were established between UseCaseData and ExoData using matching tables (e.g., if shoulder ergonomic index exceeds a threshold, recommend shoulder-support exoskeleton).		
**Ralfs, Hoffmann, and Weidner (2021)**	Setup Phase I (characterization): Identified and classified the application scenarios, user profiles, exoskeleton features (e.g., actuation, stiffness). Enabled tailored selection. Phase II (preparation): Prepared the evaluation environment, tools, and equipment. Selected the appropriate qualitative or quantitative measurement methods (electromyography, motion capture, etc.). Ensured equipment fit and functionality.	Seven-phase test protocol, experimental measurements (electromyography, kinematics), subjective ratings	Combination of qualitative and quantitative performance evaluations
	Conduct Phase III (pre-evaluation): Developed realistic test scenarios. Conducted initial trials to ensure measurement setup and methods were appropriate. Phase IV (core evaluation): Performed detailed data collection using the selected methods. Combined subjective (user feedback) and objective (biomechanics) assessments. Phase V (postevaluation): Evaluated long-term or repeated-use effects (e.g., after 4–6 weeks). Identified learning curves, fatigue adaptation, or discomfort over time.		
	Implication Phase VI (analysis): Analyzed and interpreted all collected data (qualitative and quantitative). Optionally built a structured database for comparison or simulation. Phase VII (reflection): Derived task-specific conclusions and provided practical recommendations for improvement and better integration of the exoskeletons.		
**Golabchi et al. (2023)**	Identification of need Recognized ergonomic risks or tasks with high musculoskeletal disorder (MSD) exposure in the current manufacturing process.	Organizational assessment and structured strategic framework	Organizational adoption and evaluation framework
	Stakeholder engagement Involved stakeholders from management, ergonomics, safety, human resources, and workers to ensure shared understanding and buy-in.		
	Task analysis and risk assessment Conducted detailed analysis of tasks and postures (e.g., using RULA, REBA, NIOSH lifting equation) to identify the tasks suitable for exoskeleton support.		
	Technology scouting and selection Researched available exoskeletons and preselected based on technical compatibility, target body part, task nature, and work environment.		
	Pilot testing and evaluation Performed trials of the selected exoskeleton(s) in real working conditions. Collected feedback and performance data using both subjective (e.g., comfort) and objective (e.g., electromyography) metrics.		
	Integration and training Provided structured training for users, ensured proper fitting, and integrated exoskeleton use into the work process.		
	Monitoring and continuous improvement Monitored long-term effects, updated procedures, and collected user feedback regularly to improve fit and reduce resistance.		
**This study** **(DMESAI)**	HF-FMEA Used HF-FMEA to identify the overall production process, identify the risks in different production stations, and determine the RPNs. Augmentation analysis Rated the suitability of the production tasks to ensure they were suitable for the application of exoskeletons. PROMETHEE The first stage involved rating the body part experiencing fatigue when performing production tasks. The second stage involved letting users input their preferences and select the suitable exoskeleton based on their preferences for the identified tasks	HF-FMEA, MCDM	Sequential and modular framework (follow sequence to identify risks up to the selection of a suitable exoskeleton)

## Conclusion

A decision model (DMESAI) was successfully developed in this study for exoskeleton selection in automotive production plants. The DMESAI enabled the identification of the risks of complex production tasks and assisted in the selection of a suitable exoskeleton for a particular task. The DMESAI consisted of three decision-making stages, beginning with HF-FMEA, followed by augmentation analysis, and finally, the selection of body parts experiencing fatigue and the selection of a suitable exoskeleton using Visual PROMETHEE software. The 12 criteria used for the selection of exoskeletons were rated by experts from the automotive industry using AHP. Compared with other exoskeleton selection methods, the DMESAI provided systematic ergonomic risk evaluation and enabled users to input their preferences in the selection of exoskeletons to be used in their workplace. Once the DMESAI was developed, the overall flow of the model was tested by applying tasks identified by participants from a previous survey to assess the performance of the model. Although the current application of the DMESAI is tailored for the automotive industry, the structure of the model can be adapted in other industries that require the implementation of exoskeletons. The development of a decision model for the selection of exoskeletons in the automotive industry highlights the importance of a systematic and practical decision-making method to address ergonomic challenges while enhancing the productivity in the automotive production line.

## Supporting information

S1 File Ethical application.
(PDF)

S2 File Ethical approval.
(PDF)

S3 File Raw data of manuscript.
(XLSX)
