# Supplementary material for: Development of a decision model for the selection of exoskeletons for application in automotive production plants
Source: PLoS One. 2025 Oct 14;20(10):e0333420. doi: 10.1371/journal.pone.0333420 (PMC12520347; doi:10.1371/journal.pone.0333420)
Supplement: S1 File — Ethical application. (PDF) [file pone.0333420.s001.pdf]

UNIVERSITI MALAYA

FORM 1: APPLICATION FORM

PART A: Brief Details of Project

1. Research Title : **DEVELOPMENT OF DECISION MODEL FOR THE SELECTION OF EXOSKELETON TECHNOLOGY FOR AUTOMOTIVE ASSEMBLY IN MALAYSIA**
   Project Start : January 2025

2. <u>Principal Investigator/Supervisor</u>
   Name :Raja Ariffin Bin Raja Ghazilla
   Title : Associate Professor
   Position :Associate Professor
   Telephone : 019-2665815
   Email : r_ariffn@um.edu.my
   Department :Department of Mechanical Engineering
   Academy/Faculty/Institute/Centre : Faculty of Engineering

3. <u>Co-Investigator (if any)</u>
   Name :Yap Hwa Jen
   Title : Professor
   Position :Professor
   Telephone : 012-2635646
   Email : hjyap737um.edu.my
   Department :Department of Mechanical Engineering
   Academy/Faculty/Institute/Centre : Faculty of Engineering

4. <u>Student Principal Investigator (PI) only</u>
   Name :Gan Woun Yoong
   Title : Mr.
   Position :PhD student
   Telephone : 0149653731
   Email : wygan@siswa.um.edu.my
   Department :Department of Mechanical Engineering
   Academy/Faculty/Institute/Centre : Faculty of Engineering
   Degree/Programme :PhD

5. Research funding/Grant :N/A
6. Amount of Research Grant : N/A

PART B: Data Collection

7. New data to be collected from human participant. Please tick any that apply.

☐	Focus group
☐	Experimental procedures/treatment/intervention
☐	Internet survey
☐	Observation
☒	Personal interviews
☐	Telephone survey
☐	Action research
☐	Questionnaire
☐	Others (please state):Click here to enter text.

8. Existing records with personal data.

☐	Yes
☒	No

9. Brief description of study.
   i. Background of study (less than 300words).

   In the automotive production industry, workers face demanding physical tasks that often lead to fatigue, discomfort, and work-related musculoskeletal disorders (WMSDs), particularly in repetitive or ergonomically challenging tasks. The application of exoskeletons can offer potential benefits by reducing physical strain, enhancing endurance, and improving overall task efficiency. However, the method to selecting the right and suitable exoskeleton into the workplace is still not ready.

   To address this, a decision model for exoskeleton selection has been developed, incorporating tools like Human Factor Failure Mode and Effects Analysis (HF FMEA) to identify high-risk tasks, augmentation criteria to match exoskeleton specifications with task requirements, and Visual PROMETHEE to prioritize suitable exoskeleton options. The aim of this model is to guide users in automotive production plants to choose exoskeletons that best align with specific production demands and worker needs. This interview with a production expert is crucial for validating the model’s effectiveness, gaining insights into practical applicability, and refining the model based on real-world production experiences.

ii. Rationale of study/problem statement

The integration of exoskeletons into automotive production plants is anticipated to alleviate fatigue and ergonomic challenges in tasks requiring manual operations. However, exoskeletons remain a relatively new concept for automotive production workers in Malaysia. The wide variety of exoskeleton types and models makes it challenging for potential users to determine the most suitable exoskeleton for specific tasks. This research addresses this knowledge gap by developing a framework to identify both the need for exoskeletons and the most appropriate tasks for their application. The study aims to create a decision model to assess ergonomic risks across different stations and tasks in production and to match each task with the most suitable type of exoskeleton.

iii. Objective(s) of study.

1. Understand the perception of automotive industry on the application of exoskeleton
2. Develop Exoskeleton Selection Model for automotive industry application
    - 2.2 Identify key criteria and priority
    - 2.3 Mapping criteria with exoskeleton specification
    - 2.4 Formulating and construction of decision model
3. Verification of applicability user case study

10. Study participants (new data to be collected from human participants).

i. Study sample. Please specify.

Industry Interview: The plan is to have around 30 participants that familiar with the automotive production process and work in industry.

Expertise Interview: The plan it to have around 5 participants that familiar with exoskeleton technology to comment the overall framework.

ii. How will participants be recruited? Please specify.

Industry Interview: The industry will be selected to those that are capable with the aim of the research which is the industry that conduct automotive assembly tasks, the contact to the industry will be collect through industrial introduction or through email. The participant will be recruited upon the agreement and propose by the company, research team will arrange visit and provide official letter to the company.

Expert Interview: The selection of expert will go through profile check or through the academic background of the expert to ensure they have sufficient experience in exoskeleton and the contact with either the company representative or direct contact with the academic representative through email.

The participant either from industry or from academic that have experience and knowledge in exoskeleton will be contact through email to arrange the interview.

iii. Who will perform the data collection?
The researcher name is Gan Woun Yoong

iv. Participant inclusion criteria (e.g. residents aged 18 years and above).

Industry interview:

Residency: Malaysian

Number: 30 participants

Experience: 5 years and above

Position: Executive or management level

Expert interview:

Residency: Malaysian

Number: 5 participants

Experience: 5 years and above

Position: Academic or Engineer

v. Participant exclusion criteria.

Exclude individual that are having less experience than 5 years in the related field, non-Malaysian, hold positions below the executive level in the industry, or are not academics or engineers (experts) will be excluded

vi. Are the participants given any form of payment/incentive to participate?

No.

PART C: Risk and Benefits

11. Possible benefits to participants
    Participant may gain new knowledge about the research topic. Other than that, the participant may also gain more understanding of the exoskeletons system and potential to apply into production tasks in the future. However, there is no direct benefit to the participant.

12. Risk of harm (new data to be collected from human participants).

RISK	YES	NO
Will the study involve intervention, such as action research/treatment of any type? If YES, please give details: Click here to enter text.	☐	☒
Is it possible that the duration of the procedures will cause minimal stress, in particular, for children, given their age and capacity?	☐	☒
Is it possible that the study will involve greater than minimal privacy risks, which could induce stress to research participants, such as political behaviour, illegal and sexual conduct, drug or alcohol use?	☐	☒
Will the study cause psychological stress/pain/discomfort? If YES, please state the precautions taken to minimize such stress/pain/discomfort/risk :	☐	☒
Are any of these participants from a minority/culturally identifiable/disadvantaged group? (e.g. *Orang Asli*) Please specify: Click here to enter text.	☐	☒
Will this study pose potential risk or harm to the researcher?	☐	☒

i. If any of the responses above is yes, describe potential risk/conflict of interest of the study and provide a plan to mitigate the risk/conflict of interest.

PART D: Privacy and Confidentiality

13. Describe how you will preserve participant's confidentiality as you collect and analyse the data and when you report the result
    The data of participants which include the company name, type of product, number of staff, and the position of participant, will only collect for grouping, in the analyse only will based on the outcome of the interview.

14. Existing data (if you are using existing records containing personal data).
    i. Please state the source of the data.

    ii. Are the data sensitive? (e.g. sexual preference, health status, criminal activity

☐	YES
☒	NO

    iii. Please provide full details of types of personal data to be used:

    The work-related information will be used to identify the ergonomic risk happen in the production stations such as repetitive motion in conducting tasks.

15. Data record.
    i. Describe briefly how the research data will be recorded, for example, audiotape, videotape or written notes.
    The participant will be given document to be fill in for 1st and 2nd stage of the interview, after that the feedback of the selection will be written into software of researcher.

    ii. Describe what you will do with the recorded data once it has been analysed.
    The hard-copy version of the data (e.g., questionnaires, written notes) will be stored in locked cabinets within a secured room in the university at least 5 years. Digital versions of the data will be stored in secure, password-protected computers. The archived datasets will be made available for future research purposes. Research findings will be disseminated through academic publications. The hardcopy will be fully dispose and softcopy will be deleted.

    iii. Specify who apart from yourself will have access to the research data.
    Supervisors will be granted access to ensure academic rigor, provide guidance.

iv. Details who will own the data and the results of your research.

The data belongs to the Universiti Malaya.

PART E: Conflict of Interest

16. Do any of the researchers have any potential conflicts of interest?

There is no potential conflict of interest.

PART F: Attachments

Please tick the boxes- which of the following documents are enclosed.

☒	Questionnaire/ interview script
☒	Participant Information Sheet
☒	Informed Consent Form
☐	Others : Please state :____

PART G: Declaration

"In making this application, I certify that I have read and understand the Code of Ethics and University of Malaya Manual for Responsible Research and I will comply with the ethical principles of these documents. I will submit, as appropriate, a report for amendment of an approved project, if there are significant changes to my research or if there is an adverse incident".

Signature : ______________________________

Name : Gan Woun Yoong

Date : 4/11/2024

Signature of Supervisor: ______________________________

Name : ______________________________

Date : ______________________________

Stamp : RAJA ARIFFIN RAJA GHAZILLA
Assoc . Prof
Department of Mechanical Engineering
Faculty of Engineering
Universiti Malaya

I hereby endorse that this applicant is appropriately qualified in the research area involved to conduct the proposed research project and is capable of undertaking this research study in a safe and ethical manner.

Signature : ______________________________

(Dean of Faculty /Head of Department)

Name : PROF. MADYA. DR. NURIN WAHIDAH MOHD ZULKIFLI
Ketua
Jabatan Kejuruteraan Mekanik
Fakulti Kejuruteraan
Universiti Malaya
50603 Kuala Lumpur

Date : 11/11/2024

Stamp :

UNIVERSITI MALAYA

FORM 2: PARTICIPANT INFORMATION SHEET

Basic information:

- *Researcher:*
  - *GAN WOUN YOONG (wygan@siswa.um.edu.my, 0149653731)*
  - *Department of Mechanical Engineering, Faculty of engineering*
- *Supervisor:*
  - *RAJA ARIFFIN BIN RAJA GHAZILLA,(r_ariffin@um.edu.my, 019-2665815) Associate Professor, Department of Mechanical Engineering, Faculty of Engineering;*
  - *YAP HWA JEN, Professor (hjyap737@um.edu.my, 012-2635646), Department of Mechanical Engineering,Faculty of Engineering.*

Research title :

- *DEVELOPMENT OF DECISION MODEL FOR THE SELECTION OF EXOSKELETON TECHNOLOGY FOR AUTOMOTIVE ASSEMBLY IN MALAYSIA*

Introduction :

- *This study focuses on introducing and assessing the decision model to select of exoskeletons in automotive production to help reduce physical strain and fatigue for workers performing demanding manual tasks. Exoskeletons are wearable devices designed to support the body during activities that can be physically taxing, but since this technology is new in Malaysian automotive plants, there's a need to understand which exoskeletons are best suited for specific tasks.*

  The role for researchers in this study is to develop a decision model that will help identify high-risk tasks in the production line where an exoskeleton could be beneficial. We'll analyze different tasks to assess ergonomic risks and select suitable exoskeletons to match with user perspective into suitable tasks. Through this study, we aim to guide production experts in making informed choices about integrating exoskeletons into production working environment.

Purpose :

- *To giving feedback on 4 decision model to identify the types of exoskeletons into the production environment.*
- *Giving feedback to the decision model.*

Study Procedure :

- *We will first explain the background of the study to the purpose of the study, after that will ask you to fill up the decision model framework which consists of 2 Microsoft Excel and 1 software name Visual Promethee. The Microsoft Excel consists of analysis of production station and the ergonomic risks, and the analysis of suitability of exoskeletons from selected tasks. Visual Promethee is to select the suitable exoskeleton based on user preference.*

- *This interview is expecting around 20-30 minutes.*

- *You are required to fill in information into the framework based on the practice in the automotive production and provide feedback on the preference based on the experience.*

- *This interview will be conducted by physical visit to you.*

Participation in the Study:
In order to participate in the study, you are required to:
- *Working experience in automotive production plant more than 5 years*
- *Position as executive or management level*
- *The participation in this study is completely voluntary, and we're reaching out to individuals like you who are willing and interested in contributing to our research.*

Benefit to participants:

- *You can use the framework to identify the application of exoskeleton into the production process.*
- *Certain tools are able to help the participant to identify the potential ergonomic risks in the production process.*
- *There is no direct benefit to you.*

Risk to participants:

- *With the concern of safety for the privacy data, the data will be managed properly by the researcher to ensure data safety.*
- *The study is expected to be of minimal risk and you can withdraw from the study at any time when you choose to do so and there will not be penalized for it.*

Confidentiality:

- *Video or audio taping will not be involved.*
- *Questionnaire will be collected from physical interview. Your personal information will not be publicized.*
- *Your Answer and information will be kept confidential and will not be made public unless disclosure is required by law.*
- *Signing on the consent form will authorize the review of records, analysis and use of the data arising from this study.*
- *If you have any question about the study or your rights, you can contact the researcher(s) using the contact information provided.*

Complaints

Should the participants have any concerns or questions about the research project, which they do not wish to discuss with the researchers listed in the document, then they may contact:

Reviewing REC	University of Malaya Research Ethics Committee (UMREC)
Telephone	03-79677022 (ext : 2369)
Email	umrec@um.edu.my
Mailing address	Pusat Perkhidmatan Penyelidikan (PPP)<br>Level 2,<br>Kompleks Pengurusan Penyelidikan dan Inovasi (KPPI)<br>University of Malaya

	50603 Kuala Lumpur, Malaysia

By clicking “I agree” below you are indicating that you are at least 18 years old, have read and understood this consent form and agree to participate in this research study.

☐ Agree

☐ Disagree

Please print a copy of this page for your records

UNIVERSITI MALAYA

FORM 2: LEMBARAN MAKLUMAT PESERTA

Maklumat Asas:

- *Penyelidik:*
  - *GAN WOUN YOONG (wygan@siswa.um.edu.my, 0149653731)*
  - *Jabatan Kejuruteraan Mekanikal, Fakulti Kejuruteraan*
- *Penyelia:*
  - *RAJA ARIFFIN BIN RAJA GHAZILLA,(r_ariffin@um.edu.my, 019-2665815) Professor Madya, Jabatan Kejuruteraan Mekanikal, Fakulti Kejuruteraan*
  - *YAP HWA JEN, Professor (hjyap737@um.edu.my, 012-2635646), Jabatan Kejuruteraan Mekanikal, Fakulti Kejuruteraan*

Tajuk Penyelidikan:

- *DEVELOPMENT OF DECISION MODEL FOR THE SELECTION OF EXOSKELETON TECHNOLOGY FOR AUTOMOTIVE ASSEMBLY IN MALAYSIA*

Pengenalan:

- *Kajian ini memfokuskan kepada memperkenalkan dan menilai model keputusan untuk memilih eksoskeleton dalam pengeluaran automotif bagi membantu mengurangkan tekanan fizikal dan keletihan pekerja yang melakukan tugas manual yang mencabar. Eksoskeleton adalah peranti yang boleh dipakai yang direka untuk menyokong badan semasa aktiviti yang memerlukan tenaga fizikal yang tinggi. Walau bagaimanapun, memandangkan teknologi ini masih baharu di kilang automotif Malaysia, adalah penting untuk memahami eksoskeleton mana yang paling sesuai untuk tugas tertentu.*

  Peranan penyelidik dalam kajian ini adalah untuk membangunkan model keputusan yang akan membantu mengenal pasti tugas berisiko tinggi di barisan pengeluaran yang boleh mendapat manfaat daripada penggunaan eksoskeleton. Kami akan menganalisis pelbagai tugas untuk menilai risiko ergonomik dan memilih eksoskeleton yang sesuai untuk dipadankan dengan perspektif pengguna dalam tugas yang sesuai. Melalui kajian ini, kami berharap dapat membimbing pakar pengeluaran dalam membuat keputusan yang berinformasi mengenai integrasi eksoskeleton dalam persekitaran kerja pengeluaran..

Tujuan:

- *Memberikan maklum balas terhadap 4 model keputusan untuk mengenal pasti jenis eksoskeleton yang sesuai digunakan dalam persekitaran pengeluaran.*
- *Memberikan maklum balas terhadap model keputusan tersebut..*

Prosedur Kajian:

- *Kami akan menerangkan latar belakang kajian dan tujuan kajian terlebih dahulu. Selepas itu, anda akan diminta untuk mengisi rangka kerja model keputusan yang terdiri daripada 2 fail Microsoft Excel dan 1 perisian bernama Visual*

Promethee. Fail Microsoft Excel merangkumi analisis stesen pengeluaran dan risiko ergonomik, serta analisis kesesuaian eksoskeleton berdasarkan tugas yang dipilih. Visual Promethee digunakan untuk memilih eksoskeleton yang sesuai berdasarkan keutamaan pengguna.

- *Temu bual ini dijangka mengambil masa sekitar 20-30 minit.*

- *Anda dikehendaki mengisi maklumat ke dalam rangka kerja berdasarkan amalan dalam pengeluaran automotif dan memberikan maklum balas mengenai keutamaan berdasarkan pengalaman anda.*

- *Temu bual ini akan dijalankan melalui lawatan fizikal ke tempat anda.*

Penyertaan dalam Kajian :

Untuk menyertai kajian ini, anda dikehendaki::

- *Mempunyai pengalaman kerja di kilang pengeluaran automotif selama lebih daripada 5 tahun*
- *Memegang jawatan pada peringkat eksekutif atau pengurusan.*
- *Penyertaan dalam kajian ini adalah secara sukarela sepenuhnya, dan kami menghubungi individu seperti anda yang berminat dan bersedia untuk menyumbang kepada penyelidikan kami.*

Manfaat kepada Peserta:

- *Anda boleh menggunakan rangka kerja ini untuk mengenal pasti aplikasi eksoskeleton dalam proses pengeluaran.*
- *Alat tertentu mampu membantu peserta mengenal pasti potensi risiko ergonomik dalam proses pengeluaran.*
- *Tiada manfaat langsung kepada anda.*

Risiko kepada Peserta::

- *Dengan mengambil kira keselamatan data peribadi, data akan diuruskan dengan teliti oleh penyelidik untuk memastikan keselamatan data terjamin.*
- *Kajian ini dijangka membawa risiko yang minimum, dan anda boleh menarik diri daripada kajian ini pada bila-bila masa jika anda memilih untuk berbuat demikian tanpa dikenakan sebarang penalti.*

Kerahsiaan:

- *Rakaman video atau audio tidak akan digunakan.*
- *Soal selidik akan dikumpulkan melalui temu bual secara fizikal. Maklumat peribadi anda tidak akan didedahkan kepada umum.*
- *Jawapan dan maklumat anda akan dirahsiakan dan tidak akan didedahkan kecuali jika diperlukan oleh undang-undang..*
- *Menandatangani borang persetujuan akan memberi kuasa untuk semakan rekod, analisis, dan penggunaan data yang diperoleh daripada kajian ini.*
- *Jika anda mempunyai sebarang pertanyaan mengenai kajian ini atau hak anda, anda boleh menghubungi penyelidik menggunakan maklumat hubungan yang disediakan.*

Complaints

Should the participants have any concerns or questions about the research project, which they do not wish to discuss with the researchers listed in the document, then they may contact:

Reviewing REC	University of Malaya Research Ethics Committee (UMREC)
Telephone	03-79677022 (ext : 2369)
Email	umrec@um.edu.my
Mailing address	Pusat Perkhidmatan Penyelidikan (PPP)<br>Level 2,<br>Kompleks Pengurusan Penyelidikan dan Inovasi (KPPI)<br>University of Malaya

	50603 Kuala Lumpur, Malaysia

By clicking “I agree” below you are indicating that you are at least 18 years old, have read and understood this consent form and agree to participate in this research study.

☐ Agree

☐ Disagree

Please print a copy of this page for your records

UNIVERSITI MALAYA

FORM 3: CONSENT FORM

(ENGLISH)

Title of research:

To become a participant in the research, you or your parent/legal guardian are advised to sign this Consent Form.

I herewith confirm that I have met the requirement of age and am capable of acting on behalf of myself /* as a parent/legal guardian as follows*:*

1. I understand the nature and scope of the research being undertaken.
2. All my questions relating to this research and my participation therein have been answered to my satisfaction.
3. I voluntarily agree to take part in this research, to follow the study procedures and to provide all necessary information to the investigators as requested.
4. I may at any time choose to withdraw from this research without giving reasons.
5. I have received a copy of the Subjects Information Sheet and Consent Form.
6. Except for damages resulting from negligent or malicious conduct of the researcher(s), I hereby release and discharge University of Malaya and all participating researchers from all liability associated with, arising out of, or related to my participation and agree to hold them harmless from any harm or loss that may be incurred by me due to my participation in the research.
7. I have read and understood all the terms and conditions of my participation in the research.

I have read the statements above, understand the same, and voluntarily sign this form.
Dated :_____ day _____ month ______ year

Name of participants (18 years & above): ____

Signature: ________________________ Date:_______________________

To be filled by parents/legal guardians for participants aged below 18 years

Name of parent/legal guardian: ____

Signature of parent/legal guardian: ________________ Date:_____________________

Relationship of the guardian to the participant:____

Name of researcher: _____________________________

Signature of researcher: _________________________

UNIVERSITI MALAYA

BORANG 3 : BORANG KEBENARAN
(BAHASA MELAYU)

Tajuk penyelidikan: ____

Untuk menjadi peserta dalam penyelidikan ini, anda dan ibu bapa/penjaga yang sah dinasihati untuk menandatangni Borang Kebenaran ini.

Dengan menandatangani mukasurat ini, saya mengesahkan yang berikut:

1. Saya memahami skop penyelidikan yang dijalankan.
2. Saya berpuas hati dengan semua soalan dan penglibatan saya dalam penyelidikan ini.
3. Saya secara sukarela mengambil bahagian dalam penyelidikan ini, mengikuti segala prosedur dan memberikan maklumat yang bersesuaian seperti yang diminta oleh penyelidik.
4. Saya boleh memilih untuk menarik diri daripada penyelidikan ini tanpa memberikan sebarang alasan.
5. Saya telah menerima satu salinan Borang Maklumat Peserta dan Borang Kebenaran.
6. Kecuali bagi kerosakan yang berlaku akibat daripada perlakuan cuai atau niat jahat penyelidik, saya dengan ini melepaskan penyelidik dan Universiti Malaya daripada segala tanggungan yang dikaitkan, yang timbul atau berkaitan dengan penyertaan saya serta, saya juga bersetuju untuk melepaskan penyelidik dari sebarang bahaya atau kerugian yang mungkin disebabkan oleh saya melalui penyelidikan ini.
7. Saya telah membaca dan memahami semua terma dan syarat berkenaan penglibatan saya dalam penyelidikan ini.

Saya telah membaca pernyataan di atas, memahami, dan secara sukarela menandatangi borang ini.
Tarikh : _____ hari _____ bulan ______tahun

Nama peserta (umur 18 tahun & keatas): ____

Tandatangan: ________________________ Tarikh:__________________________

Untuk diisi oleh ibu bapa/ penjaga yang sah bagi peserta berumur kurang daripada 18 tahun

Nama ibu bapa/penjaga yang sah:____

Tandatangan ibu bapa/penjaga yang sah: ______________________ Tarikh:_____________________

Hubungan penjaga dengan peserta:____

Nama Penyelidik: ____

Tandatangan Penyelidik: ____

Manual and Checklist

1. HF-FMEA
    a. Read description table ☐
    b. Identify the focus (station/tasks) ☐
    c. Selection main station from production ☐
    d. Rate the risk and count for RPN ☐
    e. Discuss for recommended controls ☐
    f. Decide processes for next stage ☐
       (optional: station/tasks/RPN/preference/control)

2. Augmentation analysis
    a. Understand the selection method and insert tasks ☐
    b. Rate the table based on selected tasks ☐
    c. Identify station/tasks for next stage ☐

3. Visual Promethee – human body selection
    a. Understand the software ☐
    b. Rate human body based on tasks selected ☐
    c. Analysis result ☐
    d. Decide exoskeleton to be rate ☐

4. Visual Promethee – exoskeleton selection
    a. Understand decision criteria ☐
    b. Rate decision criteria preference in setting (several) ☐
    c. Select relevant exoskeleton model ☐
    d. Analyse result ☐
    e. Review of relevant exoskeleton model ☐

5. Feedback form ☐

Appendix – Selection Criteria

No	Criteria	reference weight	Rating	user perspective
C1	Purchase cost	5.00%	9	5.00%
	Preference costing (RM) (range from RM6000– RM23000)			
C2	increase user's strength, endurance, and work quality	6.00%	6	10.00%
C3	reduction of WMSD and fatigue	11.00%	3	20.00%
	Preference (%, range) (range from 10% - 60%)			
C4	User friendly, comfort and safety	9.00%	4	10.00%
C5	Durability and reliability of exoskeleton	8.00%	5	10.00%
C6	Degree of freedom of exoskeleton (user flexibility)	5.00%	10	5.00%
C7	suitability for production requirement	6.00%	8	10.00%
C8	weight and size of exoskeleton	5.00%	12	10.00%
	Preference weight (kg)			kg
C9	sources of power and usage interval (battery life)	5.00%	11	10.00%
C10	maintenance and operation cost	15.00%	2	5.00%
C11	ease of maintenance and after sales support	19.00%	1	4.00%
C12	training and competency requirement	6.00%	7	1.00%
		100.00%		100.00%

	1	2	3	4
Severity (SEV)	Minor	Moderate	Major	Catastropihic
	minimal discomfort. Unlikely to cause long-term health effects.	Possible short-term discomfort but unlikely to cause serious health issues.	Could lead to musculoskeletal disorders over time.	Task causing severe pain, potential injuries, and immediate risk of musculoskeletal injury.
Occurrence (OCC)	Rare	Uncommon	Occasional	Frequent
	Occurs infrequently, only a few times a week or less.	Happens periodically, few times a day or several times a week.	Occurs frequently throughout the day, possibly multiple times an hour.	Happens continuously, with very little to no breaks in between.
Detection (DET)	High detection (easy to identify)	Medium detection	Low detection	Rare detection (almost not detectable)
	Ergonomic are promptly identified and addressed through regular inspections and feedback systems.	Ergonomic issues are noticed and addressed during assessments or when individuals report discomfort.	Ergonomic issues are often only addressed after individuals experience discomfort or report problems.	Ergonomic issues are rarely detected, and individuals continue to experience discomfort

current practice to detect

Main Tasks (station)	Sub-task	Ergonomic posture (working posture)	1 Automated / manual	Potential Ergonomics Risks	SEV	Sources of risk	OCC	Risk Control Measures	DET	RPN	RPN (Station)	Recommended controls
										0	0	
										0		
										0	0	
										0		
										0	0	
										0		
										0	0	
										0		
										0	0	
										0		
										0		
										0		
										0		
										0		

Main Tasks	Tasks	Does the task require frequent movement within station and between stations? (walking)		Does the task require repetitive movements? (to perform particular tasks)		Does the task involves lifting of heavy equipments or components?		Does the task require awkward postures (e.g., bending, twisting, squad, laydown, above shoulder, etc)?		Does the task demand high precision or complex movement and skills?		Does the task require dynamic frequent changes in direction or adjustments in posture?		Does the task require the user to hold on a position / posture for extended periods? (standing, static posture, etc)		Is the workstation sufficient to minimize WMSD among workers?		Is the working space sufficient for movement with exoskeleton?		Is the task performed in a normal environment (room temperature, not extremely hot or cold)		Is the task free from specific safety hazards (e.g., chemical exposure, risk of falling objects, high voltage, etc)?		Does the task require the contact with machinery or robots?		Does the task involves the use of tools, complex equipment or PPE that may affect the use of exoskeleton?		Does the task involves the use of vibration, impact tools, etc that may lead to fatigue and WMSD?		Does the task cause physically exhausting and pose a high risk of WMSD?		Does the workers remain in the same tasks even if it poses high WMSD?		Does the workers accept wearable device for working?		marks
		Yes - 0	No - 1	Yes - 1	No - 0	Yes - 1	No - 0	Yes - 1	No - 0	Yes - 0	No - 1	Yes - 1	No - 0	Yes - 1	No - 0	Yes - 0	No - 1	Yes - 1	No - 0	Yes - 1	No - 0	Yes - 1	No - 0	Yes - 0	No - 1	Yes - 0	No - 1	Yes - 1	No - 0	Yes - 1	No - 0	Yes - 1	No - 0	Yes - 1	No - 0	
		✓	✓	✓	✓	✓	✓	✓	✓	✓	✓	✓	✓	✓	✓	✓	✓	✓	✓	✓	✓	✓	✓	✓	✓	✓	✓	✓	✓	✓	✓	✓	✓	✓	✓	
																																				0
																																				0
																																				0
																																				0
																																				0
																																				0
																																				0
																																				0
																																				0
																																				0
																																				0

0-4 the use of exoskeleton is not priority
5-8 the application of exoskeleton can be considered
9-12 recommended for exoskeleton
13-17 strongly recommended for exoskeleton

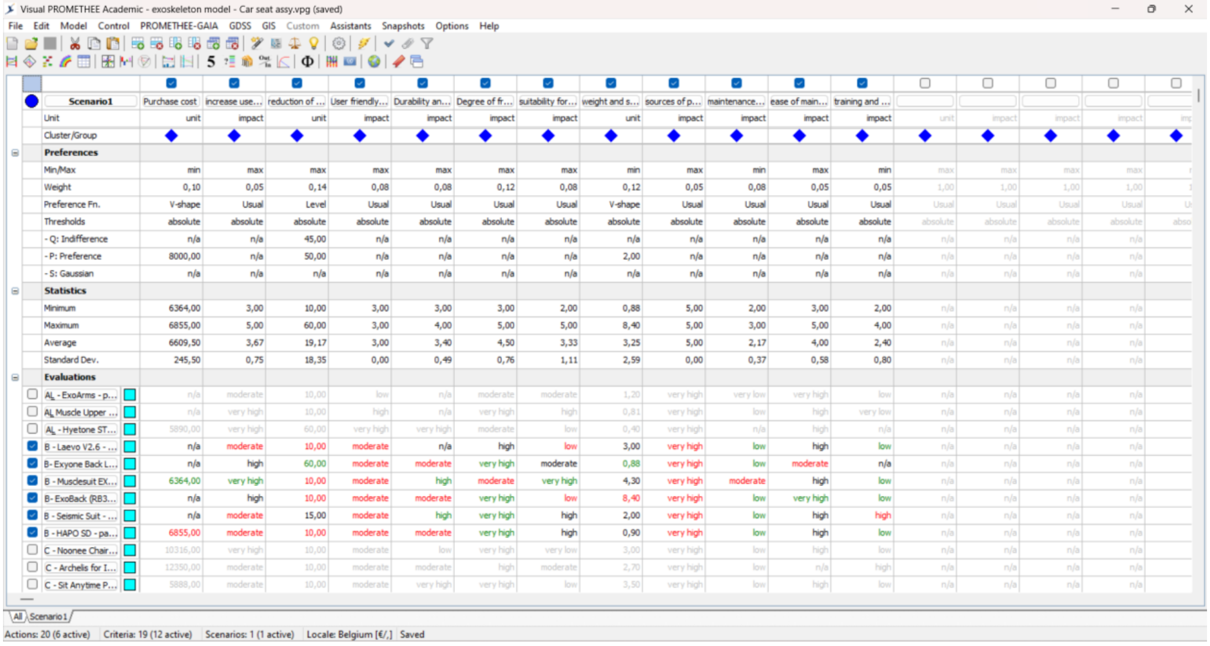

Scenario1	Purchase cost	increase use...	reduction of ...	User friendly...	Durability an...	Degree of fr...	suitability for...	weight and s...	sources of p...	maintenance...	ease of main...	training and ...				
Unit	unit	impact	unit	impact	impact	impact	impact	unit	impact	impact	impact	impact	unit	impact	impact	impact
Cluster/Group	◆	◆	◆	◆	◆	◆	◆	◆	◆	◆	◆	◆	◆	◆	◆	◆
**Preferences**																
Min/Max	min	max	max	max	max	max	max	min	max	min	max	min	max	max	max	max
Weight	0,10	0,05	0,14	0,08	0,08	0,12	0,08	0,12	0,05	0,08	0,05	0,05	1,00	1,00	1,00	1,00
Preference Fn.	V-shape	Usual	Level	Usual	Usual	Usual	Usual	V-shape	Usual	Usual	Usual	Usual	Usual	Usual	Usual	Usual
Thresholds	absolute	absolute	absolute	absolute	absolute	absolute	absolute	absolute	absolute	absolute	absolute	absolute	absolute	absolute	absolute	absolute
- Q: Indifference	n/a	n/a	45,00	n/a	n/a	n/a	n/a	n/a	n/a	n/a	n/a	n/a	n/a	n/a	n/a	n/a
- P: Preference	8000,00	n/a	50,00	n/a	n/a	n/a	n/a	2,00	n/a	n/a	n/a	n/a	n/a	n/a	n/a	n/a
- S: Gaussian	n/a	n/a	n/a	n/a	n/a	n/a	n/a	n/a	n/a	n/a	n/a	n/a	n/a	n/a	n/a	n/a
**Statistics**																
Minimum	6364,00	3,00	10,00	3,00	3,00	3,00	2,00	0,88	5,00	2,00	3,00	2,00	n/a	n/a	n/a	n/a
Maximum	6855,00	5,00	60,00	3,00	4,00	5,00	5,00	8,40	5,00	3,00	5,00	4,00	n/a	n/a	n/a	n/a
Average	6609,50	3,67	19,17	3,00	3,40	4,50	3,33	3,25	5,00	2,17	4,00	2,40	n/a	n/a	n/a	n/a
Standard Dev.	245,50	0,75	18,35	0,00	0,49	0,76	1,11	2,59	0,00	0,37	0,58	0,80	n/a	n/a	n/a	n/a
**Evaluations**																
AL - ExoArms - p...	n/a	moderate	10,00	low	n/a	moderate	moderate	1,20	very high	very low	very high	low	n/a	n/a	n/a	n/a
AL Muscle Upper ...	n/a	very high	10,00	high	n/a	very high	high	0,81	very high	low	high	very low	n/a	n/a	n/a	n/a
AL - Hyetone ST...	5890,00	very high	60,00	very high	very high	moderate	low	0,40	very high	n/a	high	n/a	n/a	n/a	n/a	n/a
B - Laevo V2.6 - ...	n/a	moderate	10,00	moderate	n/a	high	low	3,00	very high	low	high	low	n/a	n/a	n/a	n/a
B- Exyone Back L...	n/a	high	60,00	moderate	moderate	very high	moderate	0,88	very high	low	moderate	n/a	n/a	n/a	n/a	n/a
B - Musclesuit EX...	6364,00	very high	10,00	moderate	high	moderate	very high	4,30	very high	moderate	high	low	n/a	n/a	n/a	n/a
B- ExoBack (RB3...	n/a	high	10,00	moderate	moderate	very high	low	8,40	very high	low	very high	low	n/a	n/a	n/a	n/a
B - Seismic Suit - ...	n/a	moderate	15,00	moderate	high	very high	high	2,00	very high	low	high	high	n/a	n/a	n/a	n/a
B - HAPO SD - pa...	6855,00	moderate	10,00	moderate	moderate	very high	high	0,90	very high	low	high	low	n/a	n/a	n/a	n/a
C - Noonee Chair...	10316,00	very high	10,00	moderate	low	very high	very low	3,00	very high	low	high	low	n/a	n/a	n/a	n/a
C - Archelis for I...	12350,00	moderate	10,00	moderate	moderate	high	moderate	2,70	very high	low	n/a	high	n/a	n/a	n/a	n/a
C - Sit Anytime P...	5888,00	moderate	10,00	moderate	very high	very high	low	3,50	very high	low	high	low	n/a	n/a	n/a	n/a

Manual and Checklist

1. HF-FMEA
   a. Baca jadual penerangan ☐
   b. Kenal pasti fokus (stesen/tugas) ☐
   c. Pilih stesen utama daripada pengeluaran ☐
   d. Beri penilaian risiko dan kira RPN ☐
   e. Bincangkan kawalan yang disyorkan ☐
   f. Tentukan proses untuk peringkat seterusnya ☐
      (Pilihan: stesen/tugas/RPN/keutamaan/kawalan)

2. Analisis Augmentasi
   a. Fahami kaedah pemilihan dan masukkan tugas ☐
   b. Beri penilaian pada jadual berdasarkan tugas yang dipilih ☐
   c. Kenal pasti stesen/tugas untuk peringkat seterusnya ☐

3. Visual Promethee – Pemilihan Bahagian Badan Manusia
   a. Fahami perisian ☐
   b. Beri penilaian kepada bahagian badan manusia berdasarkan tugas yang dipilih ☐
   c. Analisis hasil ☐
   d. Tentukan eksoskeleton untuk dinilai ☐

4. Visual Promethee – Pemilihan Eksoskeleton
   a. Fahami kriteria keputusan ☐
   b. Beri penilaian keutamaan kriteria keputusan dalam tetapan (beberapa) ☐
   c. Pilih model eksoskeleton yang berkaitan ☐
   d. Analisis hasil ☐
   e. Semak model eksoskeleton yang berkaitan ☐

5. Feedback form ☐

Appendix – Selection Criteria

No	Criteria	reference weight	Rating	user perspective
C1	Kos Pembelian	5.00%	9	5.00%
	Julat pilihan: RM6000–RM23000			
C2	Peningkatan Kekuatan, Ketahanan, dan Kualiti Kerja Pengguna	6.00%	6	10.00%
C3	Pengurangan WMSD dan Keletihan	11.00%	3	20.00%
	Keutamaan (%): Julat 10% - 60%			
C4	Mesra Pengguna, Keselesaan, dan Keselamatan	9.00%	4	10.00%
C5	Ketahanan dan Kebolehpercayaan Eksoskeleton	8.00%	5	10.00%
C6	Darjah Kebebasan Eksoskeleton (Fleksibiliti Pengguna)	5.00%	10	5.00%
C7	Keserasian dengan Keperluan Pengeluaran	6.00%	8	10.00%
C8	Berat dan Saiz Eksoskeleton	5.00%	12	10.00%
	Keutamaan berat (kg)			kg
C9	Sumber Kuasa dan Selang Penggunaan (Hayat Bateri))	5.00%	11	10.00%
C10	Kos Penyelenggaraan dan Operasi	15.00%	2	5.00%
C11	Kemudahan Penyelenggaraan dan Sokongan Selepas Jualan	19.00%	1	4.00%
C12	Keperluan Latihan dan Kompetensi	6.00%	7	1.00%
		100.00%		100.00%

	1	2	3	4
Severity (SEV)	Kecil	Sederhana	Besar	Bencana
	Tidak mungkin menyebabkan kesan kesihatan jangka panjang	Mungkin menyebabkan ketidakselesaan sementara tetapi tidak mungkin mengakibatkan masalah kesihatan yang serius.	Mungkin membawa kepada gangguan muskuloskeletal dari masa ke masa.	Tugas yang menyebabkan kesakitan teruk, kecederaan berpotensi, dan risiko segera kecederaan muskuloskeletal.
Occurrence (OCC)	Jarang	Tidak Lazim	Kadang-kadang	Kerap
	Berlaku jarang, hanya beberapa kali seminggu atau kurang.	Berlaku secara berkala, beberapa kali sehari atau beberapa kali seminggu.	Berlaku kerap sepanjang hari, mungkin beberapa kali dalam sejam.	Berlaku secara berterusan, dengan sedikit atau tiada rehat antara kejadian.
Detection (DET)	Pengesanan Tinggi	Pengesanan Sederhana	Pengesanan Rendah	Pengesanan Jarang
	Isu ergonomik dikenalpasti dan ditangani segera melalui pemeriksaan berkala dan sistem maklum balas.	Isu ergonomik diperhatikan dan ditangani semasa penilaian atau apabila individu melaporkan ketidakselesaan.	Isu ergonomik sering hanya ditangani selepas individu mengalami ketidakselesaan atau melaporkan masalah.	Isu ergonomik jarang dikesan, dan individu terus mengalami ketidakselesaan tanpa intervensi.

current practice to detect

Tugas Utama (Stesen)	Sub-Tugas	Postur Ergonomik (Postur Kerja)	Automasi / Manual	Potensi Risiko Ergonomik	SEV	Sumber Risiko	OCC	Langkah Kawalan Risiko	DET	RPN	RPN (Station)	Kawalan yang Disyorkan
										0	0	
										0		
										0	0	
										0		
										0	0	
										0		
										0	0	
										0		
										0	0	
										0		
										0		
										0		
										0		
										0		

Tugas Utama (Stesen)	Sub-Tugas	Adakah tugas memerlukan pergerakan kerap di dalam stesen dan antara stesen? (berjalan kaki)		Adakah tugas memerlukan pergerakan berulang? (untuk melaksanakan tugas tertentu)		Adakah tugas melibatkan pengangkatan peralatan atau komponen yang berat?		Adakah tugas memerlukan postur yang janggal? (contoh: membongkok, memusing,		Adakah tugas memerlukan ketepatan tinggi atau pergerakan dan kemahiran yang kompleks?		Adakah tugas memerlukan perubahan arah atau penyesuaian postur yang kerap dan dinamik?		Adakah tugas memerlukan pengguna mengekalkan kedudukan/postur untuk tempoh masa		Adakah stesen kerja mencukupi untuk meminimumkan Risiko Gangguan Muskuloskeletal Pekerja (WMSD)?		Adakah ruang kerja mencukupi untuk pergerakan bersama eksoskeleton?		Adakah tugas dilaksanakan dalam persekitaran biasa? (suhu bilik, tidak terlalu panas atau sejuk)		Adakah tugas bebas daripada bahaya keselamatan tertentu? (contoh: pendedahan kimia, risiko objek jatuh,		Adakah tugas memerlukan sentuhan dengan mesin atau robot?		Adakah tugas melibatkan penggunaan alat, peralatan kompleks, atau PPE yang mungkin		Adakah tugas melibatkan penggunaan alat bergetar, alat hentakan, dan sebagainya yang		Adakah tugas menyebabkan keletihan fizikal yang tinggi dan menimbulkan risiko WMSD yang tinggi?		Adakah pekerja kekal menjalankan tugas yang sama walaupun ia menimbulkan risiko WMSD yang tinggi?		Adakah pekerja menerima penggunaan peranti boleh pakai (wearable device) untuk bekerja?		markah
		Yes - 0	No - 1	Yes - 1	No - 0	Yes - 1	No - 0	Yes - 1	No - 0	Yes - 0	No - 1	Yes - 1	No - 0	Yes - 1	No - 0	Yes - 0	No - 1	Yes - 1	No - 0	Yes - 1	No - 0	Yes - 1	No - 0	Yes - 0	No - 1	Yes - 0	No - 1	Yes - 1	No - 0	Yes - 1	No - 0	Yes - 1	No - 0	Yes - 1	No - 0	
		✓	✓	✓	✓	✓	✓	✓	✓	✓	✓	✓	✓	✓	✓	✓	✓	✓	✓	✓	✓	✓	✓	✓	✓	✓	✓	✓	✓	✓	✓	✓	✓	✓	✓	
																																				0
																																				0
																																				0
																																				0
																																				0
																																				0
																																				0
																																				0
																																				0
																																				0
																																				0

0-4 the use of exoskeleton is not priority
5-8 the application of exoskeleton can be considered
9-12 recommended for exoskeleton
13-17 strongly recommended for exoskeleton
