# Supplementary material for: Development of a decision model for the selection of exoskeletons for application in automotive production plants
Source: PLoS One. 2025 Oct 14;20(10):e0333420. doi: 10.1371/journal.pone.0333420 (PMC12520347; doi:10.1371/journal.pone.0333420)
Supplement: S2 File — Ethical approval. (PDF) [file pone.0333420.s002.pdf]

UNIVERSITI MALAYA

UM.TNC 2/UMREC

22 January 2025

Gan Woun Yoong
Faculty of Engineering
Universiti Malaya

Dear Sir/Madam,

RESEARCH ETHICS CLEARANCE APPLICATION

On behalf of the Universiti Malaya Research Ethics Committee (UMREC), we are pleased to inform you that your application as detailed below has been approved:

**Name of Student PI:**	**Gan Woun Yoong**
**Title of Proposal:**	**DEVELOPMENT OF DECISION MODEL FOR THE SELECTION OF EXOSKELETON TECHNOLOGY FOR AUTOMOTIVE ASSEMBLY IN MALAYSIA**
**Reference Number:**	**UM.TNC2/UMREC_4200**

Kindly proceed with the research in accordance with the Universiti Malaya Research Ethics Guidelines and please do take note that this approval is valid from **January 2025 till January 2028.**

Please be informed that research ethics audit may take place to ensure that all approved applications are being conducted in keeping with conditions of approval of the reviewing committee.

In the case of amendment to the research project, please complete the "Amendment Form" (available from the website) and submit to UMREC office for approval.

For more information about Universiti Malaya Research Ethics, please visit: https://umresearch.um.edu.my/research-ethics/

Thank you.

Home of the Bright, Land of the Brave
Di Sini Bermulanya Pintar, Tanah Tumpahnya Berani

Yours sincerely,

ASSOC. PROF. DR. RAIDA BINTI ABU BAKAR
Chairperson,
Universiti Malaya Research Ethics Committee (Non-Medical)

c.c
Deputy Dean (Research), Faculty of Engineering
Universiti Malaya

UNIVERSITI MALAYA RESEARCH ETHICS COMMITTEE (UMREC NON-MEDICAL)
Universiti Malaya, Lembah Pantai, 50603 Kuala Lumpur
Tel No.: 03-7967 2369 • E-mail: umrec@um.edu.my • https://umresearch.um.edu.my/research-ethics/
